# Eggshell Membrane in Gut Health: Processing-Dependent Redox, Barrier, Immune, and Microbiome Effects

**DOI:** 10.3390/foods15203673

**Published:** 2026-10-16

**Authors:** Aslı Çakır, Tamer A. E. Ahmed, Maxwell T. Hincke

**Affiliations:** 1 Department of Cellular and Molecular Medicine, Faculty of Medicine, University of Ottawa, Ottawa, ON K1H 8M5, Canada; acaki042@uottawa.ca (A.Ç.); tahmed@uottawa.ca (T.A.E.A.); 2 Department of Innovation in Medical Education, Faculty of Medicine, University of Ottawa, Ottawa, ON K1H 8M5, Canada

**Keywords:** eggshell membrane, gut health, oxidative stress, processing, intestinal barrier, gut microbiota, functional foods

## Abstract

Gut health is maintained through interactions among intestinal barrier integrity, microbial homeostasis, host-immune function, and redox balance. Eggshell membrane (ESM), a protein-rich poultry by-product, contains structural components and derived peptides with antioxidant and anti-inflammatory activity in experimental systems. Increasing evidence indicates that ESM preparations can also influence epithelial barrier responses, intestinal inflammation, microbial community composition, and microbial metabolites. Because native, micronized, hydrolyzed, fractionated, and formulated ESMs differ in physical and chemical properties, processing is likely to influence gastrointestinal fate and biological activity. This review integrates chemical, cellular, animal, and human evidence to connect preparation-dependent properties with gastrointestinal exposure and intestinal redox, barrier, immune, and microbiome responses. It also considers oral delivery, dose, safety, and experimental approaches needed to resolve mechanism and translation. The evidence supports a biologically coherent intestinal-health framework for ESM, while controlled human studies are still needed to establish clinically meaningful gut-health effects and an effective intestinal dose.

## 1. Introduction

### 1.1. Conceptual Framework of Gut Health and Redox Homeostasis

Gut health is maintained through coordinated regulation of the intestinal barrier, microbiota, immune system, host metabolism, and redox balance. Moreover, the microbiota contributes to this homeostasis by shaping mucus production, metabolic activity, immune signaling, and resistance to pathogen colonization [1,2,3]. These processes are closely linked rather than functioning independently.

In addition, reactive oxygen species (ROS) participate in the regulation of gut health. At controlled concentrations, ROS participate in epithelial and immune signaling, antimicrobial defense, proliferation, and tissue repair [4,5,6]. However, when ROS production exceeds endogenous antioxidant capacity, oxidative stress impairs epithelial integrity and promotes inflammation [7]. Therefore, gut health should be considered a dynamic equilibrium where physiological redox signaling is preserved while excess oxidative stress is controlled. Dysbiosis, also defined as an altered composition or function of the gut microbial community, contributes to oxidative stress, while changes in epithelial metabolism and oxygen availability alter the microbial community [3,8].

The interconnected framework helps explain the interest in eggshell membrane (ESM), which is a food-derived biomaterial associated with antioxidant, anti-inflammatory, epithelial barrier, and microbiome-related effects [9,10,11]. These effects have been reported across different ESM preparations and experimental models, including changes in glutathione-related defenses, epithelial restitution, inflammatory mediators, gut microbiota composition, and microbial metabolites [11,12,13]. However, the strength of evidence differs among these outcomes. This makes it important to understand whether specific ESM preparations can produce meaningful effects in the intestine and which mechanisms are involved.

### 1.2. ESM Rationale, Review Scope and IBD as a Mechanistic Model

Eggshell membrane is a poultry-industry by-product with a fibrous matrix containing proteins, collagens, glycosaminoglycans (GAGs), and other potentially bioactive components [9,14,15]. Evidence from one ESM preparation cannot automatically be generalized to another. Mechanical milling changes particle size, whereas hydrolysis and subsequent fractionation alter the recovered protein and peptide profiles. Preparations can differ in solubility, susceptibility to digestion, and measured biological activity [16,17,18,19,20]. These properties require separate characterization rather than inference from a processing label. Most evidence related to gut health remains preclinical, and the active components, gastrointestinal fate, and effective intestinal dose remain unclear [10,11,16,21,22].

Previous reviews have described ESM composition, separation and solubilization technologies, and broader biomaterial, functional-food, and nutraceutical applications [9,23,24]. The specific contribution of this review is to connect preparation-dependent material properties and gastrointestinal fate with the strength of evidence for intestinal redox, barrier, immune, and microbiome responses. Native, micronized, hydrolyzed, and fractionated ESMs are considered separately. Experimentally measured properties and responses are distinguished from hypotheses about regional intestinal exposure and microbial mediation, identifying which links require direct comparison or causal testing.

The reported ESM effects concern processes that also interact during intestinal inflammation, providing a mechanistic reason to examine inflammatory bowel disease (IBD). In metabolically stressed epithelial models and human colonic tissue examined ex vivo, mitochondrial ROS contributed to increased bacterial passage across the epithelium [25]. Separately, microbiota-dependent epithelial PPAR-γ signaling was shown to limit the availability of respiratory electron acceptors and restrain Enterobacteriaceae expansion [3]. Longitudinal human multi-omics studies also associate IBD activity with changes in microbial composition and metabolism [26]. These findings connect redox imbalance, barrier injury, inflammation, and microbial dysfunction, providing a framework for examining ESM-associated responses without implying that ESM has been shown to modify each pathway.

This choice is also supported by direct ESM research. Fine ESM powder and ESM hydrolysates have been investigated in dextran sulfate sodium (DSS)-induced colitis, and dietary ESM has been studied in interleukin-10-deficient mice [11,13,22]. These models allow epithelial injury, inflammatory responses, and microbial changes to be evaluated within the same experimental setting. Nevertheless, IBD does not represent all aspects of gut health, and experimental colitis does not reproduce the full complexity of human disease. Therefore, these studies are used to examine possible mechanisms, alongside other intestinal models and healthy-adult studies, rather than to establish clinical efficacy for IBD.

### 1.3. Review Methodology

This narrative review was developed through iterative literature searches conducted primarily in PubMed, Scopus, Google Scholar, and ScienceDirect. The literature search was initiated on 10 June 2026, and continued throughout manuscript preparation, with the final search update performed on 8 September 2026. Rather than using a single systematic search query, searches were conducted according to the major topics addressed in this review, including ESM composition and processing, antioxidant activity, intestinal barrier function, inflammatory responses, gut microbiota and microbial metabolites, experimental intestinal-disease models, human evidence, and translational considerations.

Search terms were used in different combinations and included eggshell membrane, ESM, eggshell membrane hydrolysate, eggshell membrane peptides, gut health, intestinal barrier, gut microbiota, gut microbiome, inflammatory bowel disease, ulcerative colitis, Crohn’s disease, oxidative stress, antioxidant activity, Nrf2, NF-κB, short-chain fatty acids, digestion, hydrolysis, processing, and oral delivery. Representative searches included combinations such as (“eggshell membrane” OR ESM) AND (intestinal OR “gut health” OR microbiota OR microbiome); (“eggshell membrane” OR ESM) AND (“oxidative stress” OR antioxidant OR glutathione OR Nrf2); (“eggshell membrane” OR “eggshell membrane hydrolysate”) AND (digestion OR hydrolysis OR processing OR gastrointestinal); and (“eggshell membrane” OR ESM) AND (colitis OR “inflammatory bowel disease” OR DSS). Additional targeted searches were conducted for specific mechanisms and the topics discussed throughout this review.

In vitro, animal, and human studies were considered to represent different levels of ESM evidence. ESM-specific studies that address composition, processing, digestibility, antioxidant activity, gut or immune responses, microbiota, safety, or human use were included. Non-intestinal ESM studies were not considered direct evidence of intestinal efficacy but were retained selectively when they provided relevant information on antioxidant mechanisms, processing, safety, or oral tolerability. Supporting non-ESM studies were included when needed to establish the mechanistic background for intestinal redox regulation, epithelial barrier function, immune responses, microbiome activity, IBD, or delivery strategies.

Recent ESM-specific publications, specifically those from the preceding 10 years, were prioritized. However, older studies were retained when they provided foundational or directly relevant evidence. Reference lists of relevant primary studies and review articles were also examined to identify additional publications.

## 2. Eggshell Membrane: Composition, Processing and Gastrointestinal Fate

### 2.1. Structural and Biochemical Composition of ESM

Eggshell membrane is a fibrous biomaterial containing collagen-like proteins, GAGs, and other proteins with potential biological activity [9,14,15,27]. It is also a by-product of the poultry industry that is often treated as industrial waste despite its potential as a nutraceutical and functional food ingredient [10,27]. Structurally, ESM consists primarily of inner and outer fibrous membrane layers that are located beneath the mineralized eggshell. Membrane thickness also differs between the two layers, with the outer layer measuring about 50–70 μm and the inner layer 15–26 μm [28].

ESM is predominantly proteinaceous, and proteomic studies have identified hundreds of constituent proteins [9]. Collagens account for roughly 10% of the membrane and include types I, V, and X [14,29,30]. These collagen types contribute to the fibrous structural organization of ESM. ESM also contains hyaluronic acid, sulfated GAGs, ovotransferrin, lysozyme, and ovocalyxin-36 [9,15,27]. Additional constituents include fibronectin and osteopontin, along with sialic acid, uronic acid, and low levels of saccharides [9].

The inner and outer fibrous layers share major constituents but are not chemically identical. Nakano et al. [31] found higher sialic acid concentrations in the inner membrane, whereas nitrogen and uronic acid concentrations were similar between layers. Earlier biochemical and immunohistochemical work detected type I- and type V-like collagen in both layers, with their distribution also varying between coarse and fine fibers [32]. Thus, the layers should not be described as having separate collagen compositions. These results identify differences in selected constituents, but they do not provide a complete quantitative comparison of the two-layer proteomes.

Layer composition could influence the components recovered if a separation method preferentially retains one layer or associated shell material. This is a plausible source of variation, rather than an established explanation for differences in intestinal activity. In the gut-health studies discussed here, ESM preparations were not compared using separately isolated inner and outer layers. Hydrolysis and fractionation can also change the accessibility and recovery of components from the combined membrane [16,18,27]. Consequently, current evidence cannot assign processing-dependent antioxidant, epithelial, or microbial effects to a particular membrane layer. Direct comparisons of layer composition, processing yield, and biological activity would be needed to test that relationship.

### 2.2. Processing, Digestibility and Gastrointestinal Bioaccessibility

Solubility describes the amount or fraction of material that enters solution under specified conditions, including pH, temperature, and extraction medium. Digestibility describes its susceptibility to gastrointestinal breakdown, assessed through peptide generation or an appropriately defined in vivo digestibility measurement. Solubilization itself does not demonstrate peptide-bond cleavage, and a smaller physical particle is not necessarily a smaller protein or peptide. These distinctions are important when comparing processed ESM preparations [17,19,20].

Native ESM has a densely cross-linked fibrous structure, with disulfide bonds and lysine-derived crosslinks contributing to its poor solubility [23]. In a rat nitrogen-balance study, ESM protein had a fecal nitrogen-based digestibility of 87.0%, compared with 94.8% for its enzymatic hydrolysate and 98.5% for casein [17]. These preparation-specific, whole-tract measurements show lower digestibility relative to casein; they do not mean that native ESM is wholly indigestible. They also do not quantify intact bioactive-peptide absorption or determine how much material reaches the colon. The results from such in vivo measurements should be distinguished from soluble-protein recovery and peptide profiles measured during simulated digestion.

Hydrolysis can increase the recovery of soluble components and release peptides, although its effects depend on the method and preparation. Keratinase treatment of ESM yielded collagen peptides and measurable GAGs, including a high-molecular-weight polysaccharide fraction consistent with hyaluronic acid [27]. Enzymatic ESM hydrolysates have also reduced ROS and IL-8 secretion and increased intracellular glutathione in Caco-2 cells [16]. While these findings demonstrate the cellular activity of the tested hydrolysates, solubility, gastrointestinal digestibility, and intestinal efficacy should be evaluated separately.

However, hydrolyzed ESM preparations differ across studies. In one protocol, ESM was first treated under alkaline conditions using 0.6 M NaOH and 10% ethanol at 55 °C for 2 h. After adjustment to pH 10, the material underwent a 4 h digestion with 1% Alcalase, followed by a 12 h treatment with 2% Protease A, N, P, or S [16]. The Alcalase–Protease S hydrolysate was then separated by molecular size into <5, 5 to 10, and >10 kDa fractions, which showed different antioxidant activities. Another study used 5% Alcalase at pH 8.0 and 55 °C and examined hydrolysis over 2 to 14 h [21]. A different approach used a thermostable keratinase with sodium sulfite at pH 8.0 and 55 °C for 3 h, resulting in more than 85% ESM decomposition [27]. Differences in enzyme system, chemical pretreatment, pH, reaction time, and peptide fractionation complicate direct comparisons. Fractionation can enrich material with a particular molecular-weight range, but the most active fraction depends on the endpoint; greater activity in one antioxidant assay does not establish greater overall intestinal benefit [12,16,33].

Fine or micronized ESM is produced by mechanical particle-size reduction. This increases exposed surface area without necessarily cleaving the proteins in the matrix; therefore, its main potential advantage is improved physical access to the material without a separate hydrolysis step. Its limitation is that cross-linked proteins can remain poorly soluble and resistant to digestion. In a modified INFOGEST-based study, micronized ESM generated fewer small peptides than an ESM hydrolysate under the tested conditions [19]. Fine ESM nevertheless showed epithelial and inflammatory effects in experimental colitis [11]. These findings justify studying milled preparations, but do not establish that reducing particle size caused the biological effects or that finer particles are always more effective.

Jet-O-mized ESM (JEM) is specifically produced by jet milling and should be distinguished from hydrolyzed JEM. Younes et al. reported a mean JEM particle size of 5.7 μm, followed by KOH-mediated hydrolysis to generate whole and soluble hydrolysate fractions [18]. The combined process offers a route to soluble bioactive material, with antioxidant and cellular effects reported in vitro. Limitations include the need for neutralization and desalting, incomplete disclosure of optimized hydrolysis conditions, and absence of demonstrated intestinal efficacy in vivo. Because milling and hydrolysis were combined, the observed functional gains cannot be attributed to jet milling alone [18].

Bioaccessibility refers to the fraction released from a matrix during digestion and available for absorption or contact with intestinal targets. Systemic bioavailability additionally requires absorption and subsequent delivery to circulation, and it cannot be inferred from solubility or antioxidant activity after simulated digestion. Unabsorbed material can remain available for distal intestinal and microbial interactions, but its amount and biological activity must be measured [34]. Table 1 summarizes the processing-dependent characteristics of different ESM preparations.

### 2.3. Gastrointestinal Stability and Principles of Oral Delivery

Orally administered bioactives encounter changes in pH, digestive enzymes, bile salts, and interactions with the food matrix. Digestion can destroy active peptide sequences or release smaller fragments with different properties. Thus, the aim of a delivery system is to preserve and release the relevant active material at its intended intestinal site, rather than to maximize solubility or systemic absorption [35,36].

The distinction between solubility and digestibility remains important during this process. A comparison of three commercial ESM preparations found markedly different protein solubilities, yet limited peptide generation during the simulated gastrointestinal digestion used in that study [20]. A soluble preparation can remain resistant to further proteolysis. Conversely, the lower fecal digestibility of native ESM relative to casein in rats does not establish complete resistance to gastrointestinal breakdown [17]. These findings are not interchangeable because the preparations, models, and analytical endpoints differ.

ESM administered without a designed carrier can still produce local biological effects. Released peptides or other components may interact with the intestinal epithelium, while material that escapes upper-gastrointestinal digestion may remain available for microbial metabolism. Systemic absorption is not required for a luminal action, although access to subepithelial targets requires additional transport [36]. Unencapsulated ESM powder and hydrolysates have produced effects in animal models [11,13,22]. Their advantage is a simpler formulation; their limitation is the lack of designed control over the timing and site of release and protection of sensitive components during transit.

Compared with unencapsulated material, a suitable carrier can reduce premature exposure to gastric conditions and digestive enzymes, delay release until an intestinal pH or microbial-enzyme trigger is reached and sustain local exposure. These features may preserve an active peptide sequence and increase the amount reaching the intended site. Polysaccharide capsules, hydrogels, and nanoparticles provide examples of protection and controlled release in other bioactive systems [37,38,39]. However, performance depends on the cargo and carrier; these advantages cannot be assumed for every ESM preparation. A carrier designed to protect a labile peptide may be less useful for a preparation whose intended action depends on contact of the unencapsulated matrix with the microbiota.

ESM-specific studies provide initial support for these formulation benefits. Carboxymethyl chitosan nanoparticles carrying ESM peptides showed pH-responsive release and better preservation of antioxidant activity during simulated gastrointestinal digestion than free peptides [35]. In a separate study, chitosan/fucoidan nanoparticles containing soluble ESM protein remained stable at simulated gastric pH and released protein under intestinal conditions; the formulation was also examined in an epithelial–macrophage co-culture model [40]. These experiments support gastrointestinal protection and controlled release in vitro. They do not establish improved ESM exposure or efficacy in human IBD, and the biological activity of the carrier itself must be considered.

Formulation also introduces additional processing steps and may limit release if the carrier retains the active material too strongly. Its value should be tested against an equal dose of the same unencapsulated preparation and a blank-carrier control. Relevant comparisons include peptide recovery after digestion, release at the intended site, local exposure, and epithelial, inflammatory, or microbial outcomes. A benefit in stability or release should be distinguished from a demonstrated improvement in biological efficacy.

### 2.4. ESM Processing, Formulation and Gastrointestinal Availability

**Figure 1:**
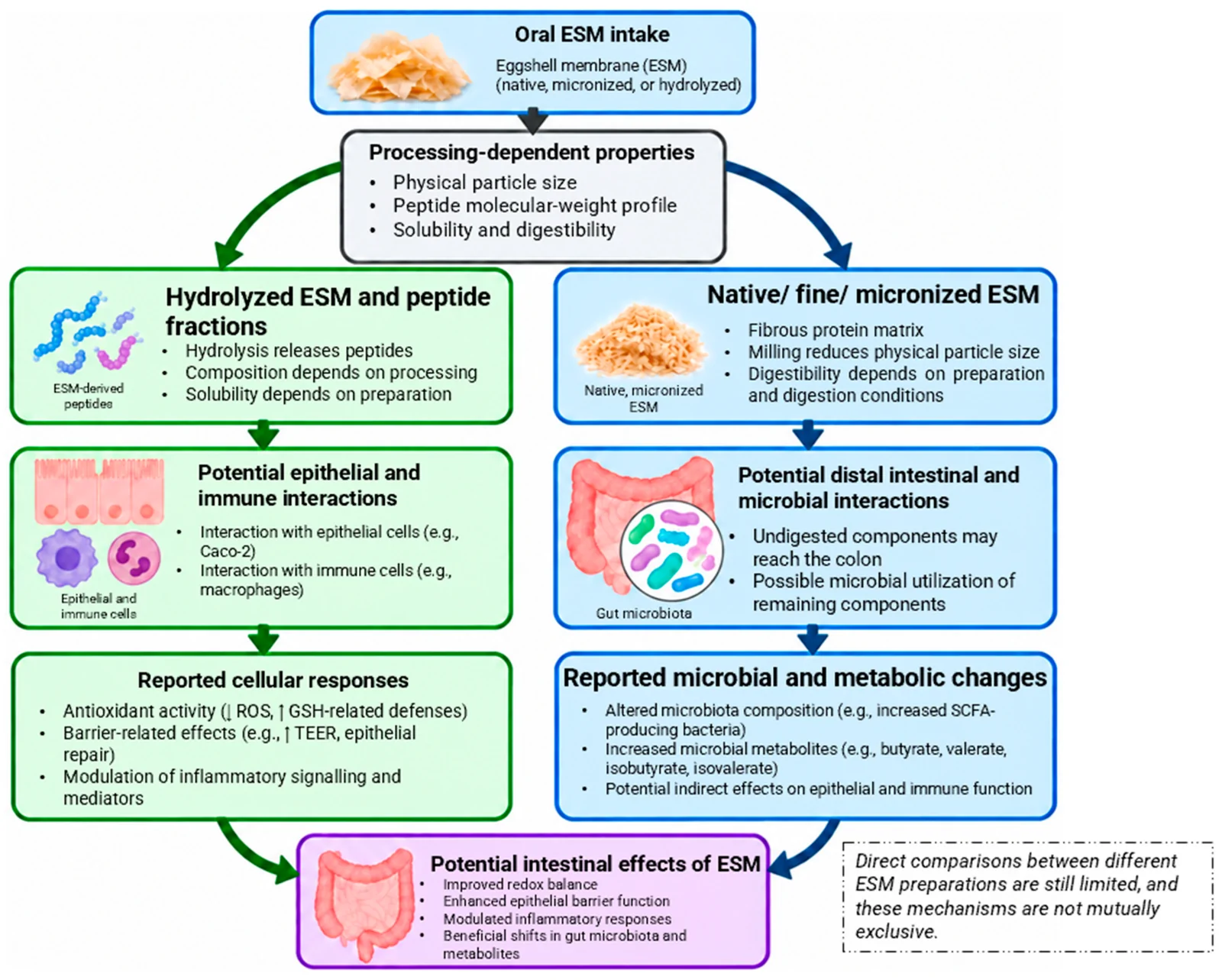
Preparation-dependent properties and proposed intestinal interactions of eggshell membrane (ESM). Processing alters physical and chemical properties that can influence gastrointestinal availability. Reported epithelial, immune, microbial, and metabolite responses are shown alongside proposed exposure routes; the routes are not mutually exclusive and require direct comparison in matched preparations.

Processing and formulation can alter the physical form, peptide profile, stability, and gastrointestinal availability of ESM. These differences provide a plausible basis for preparation-dependent intestinal responses. Figure 1 integrates the measured material properties and reports cellular or microbial responses with the gastrointestinal exposure routes proposed from current evidence.

Direct comparative studies remain a major need. Native, micronized, hydrolyzed, fractionated, and formulated preparations should be compared using the same starting material and matched doses, with measurements of particle size, soluble-protein recovery, peptide profiles, digestion, regional intestinal exposure, and biological responses. Such studies would determine which processing steps improve delivery of active ESM components and which are most relevant to epithelial, immune, or microbial targets.

### 2.5. Safety, Standardization and Regulation

Available studies suggest that ESM is generally safe, although safety may vary depending on the specific preparation and processing method used. Repeated oral administration of NEM at doses up to 2000 mg/kg/day produced no treatment-related toxic effects in rats [41]. EFSA also concluded that a specific enzymatically hydrolyzed egg membrane preparation was safe at intakes up to 500 mg/day for adults, excluding pregnant and lactating women [42]. However, ESM is derived from egg, and the EFSA assessment also considered residual egg proteins and possible allergenicity associated with papain used during processing [42].

Standardization is important because ESM composition and properties can vary with the source and processing method, which influence protein structure, solubility, recovery, and the final product composition [23,24]. Therefore, studies should report on the membrane source, separation and purification method, particle size, hydrolysis conditions, protein content, molecular-weight distribution, and relevant components. Residual minerals, processing agents, allergens, microbial contamination, and differences between batches should also be considered [24,42]. Careful characterization is crucial to account for differences among ESM products and to support meaningful comparison across studies.

Regulatory requirements depend on the product, its intended use, and the claims being made. This is illustrated by the EFSA assessment of egg membrane collagen peptides, which evaluated a defined preparation, production process, composition, intended population, and maximum daily intake under the EU novel-food framework [42]. Any stronger health claims would require further human evidence and safety data.

## 3. Mechanistic Background for ESM in Intestinal Health

### 3.1. Redox–Barrier–Immune–Microbiome Axis in Intestinal Homeostasis and Disease

#### 3.1.1. Gastrointestinal and Epithelial Context

The intestinal epithelium separates luminal contents from underlying tissues and responds to dietary and microbial products [43]. The small intestine supports digestion and absorption, whereas the colon provides a major site of microbial fermentation [44,45]. These regional functions motivate investigation of ESM gastrointestinal fate, but they do not establish where a particular preparation acts.

Epithelial renewal, mucus, and intercellular junctions maintain the intestinal barrier [46,47,48,49]. ESM-associated epithelial restitution in DSS-treated mice and changes in Caco-2 transepithelial electrical resistance (TEER) therefore make repair and barrier function directly relevant endpoints [11,18]. Tight-junction proteins such as claudins, occludin, and zonula occludens regulate paracellular permeability, while mucus limits microbial contact with the epithelium [47,48,49,50,51,52,53,54]. Functional measurements such as TEER, permeability, histology, and restitution are most informative when interpreted together with molecular markers.

#### 3.1.2. Physiological Redox Signaling and Endogenous Antioxidant Defense

Physiological ROS signaling supports epithelial renewal and host defense, whereas excessive oxidative stress can impair intestinal barrier function [4,5,6,7,55]. NOX1-dependent ROS production participates in epithelial proliferation and wound repair, selected intestinal bacteria can induce Nrf2-dependent cytoprotective responses, and activated inflammatory cells provide additional sources of reactive species [4,5,6,55,56]. These contexts make both oxidative injury and endogenous antioxidant responses relevant to the interpretation of ESM activity.

Superoxide dismutases, catalase, glutathione peroxidases, and glutathione contribute to cellular antioxidant defense [57,58,59,60,61,62,63]. ESM-derived peptide fractions have increased glutathione and glutathione-related enzyme activities in stressed Caco-2 cells [12]. Nrf2–Keap1 is an important regulator of antioxidant gene expression [64,65,66,67,68] and provides a mechanistic hypothesis for these responses, although pathway activation should be assigned only when it is measured directly.

#### 3.1.3. Intestinal Immune–Microbiota Interactions

Physiological Toll-like receptor and MyD88 signaling contributes to epithelial homeostasis and antibacterial defense, while IL-22-producing innate lymphoid populations support epithelial repair and antimicrobial responses [69,70,71]. These interactions link microbial recognition with host defense. For ESM, reported changes in inflammatory mediators should be evaluated alongside epithelial and microbial outcomes, while distinguishing controlled cell exposure from oral administration.

Microbial composition varies among healthy individuals, and taxonomically different communities can share metabolic functions [72,73]. Fecal samples also do not represent every intestinal region [74]. Consequently, changes in alpha diversity or individual bacterial groups after ESM supplementation require interpretation alongside metabolites, host responses, and functional measurements.

#### 3.1.4. Microbial Metabolites and Integrated Homeostasis

Butyrate links microbial activity to colonocyte metabolism, barrier regulation, and immune responses [3,75,76,77]. Changes in cecal organic acids after dietary ESM therefore provide useful functional context for microbiota findings [78,79]. However, accompanying microbial and metabolite changes do not establish that ESM-derived substrates produced those metabolites or that the metabolites mediated the host response.

#### 3.1.5. Convergence of Oxidative Stress, Barrier Dysfunction, Inflammation and Dysbiosis

Oxidative stress, epithelial injury, microbial exposure, and inflammatory signaling form an interconnected network in intestinal disease [3,7,43,55,80,81,82,83,84,85]. Within this network, mitochondrial dysfunction can amplify ROS production and impair epithelial repair, while inflammation can alter epithelial metabolism and the microbial environment. This framework provides a biological context for integrating the redox, barrier, immune, and microbiome responses reported in ESM studies.

**Figure 2:**
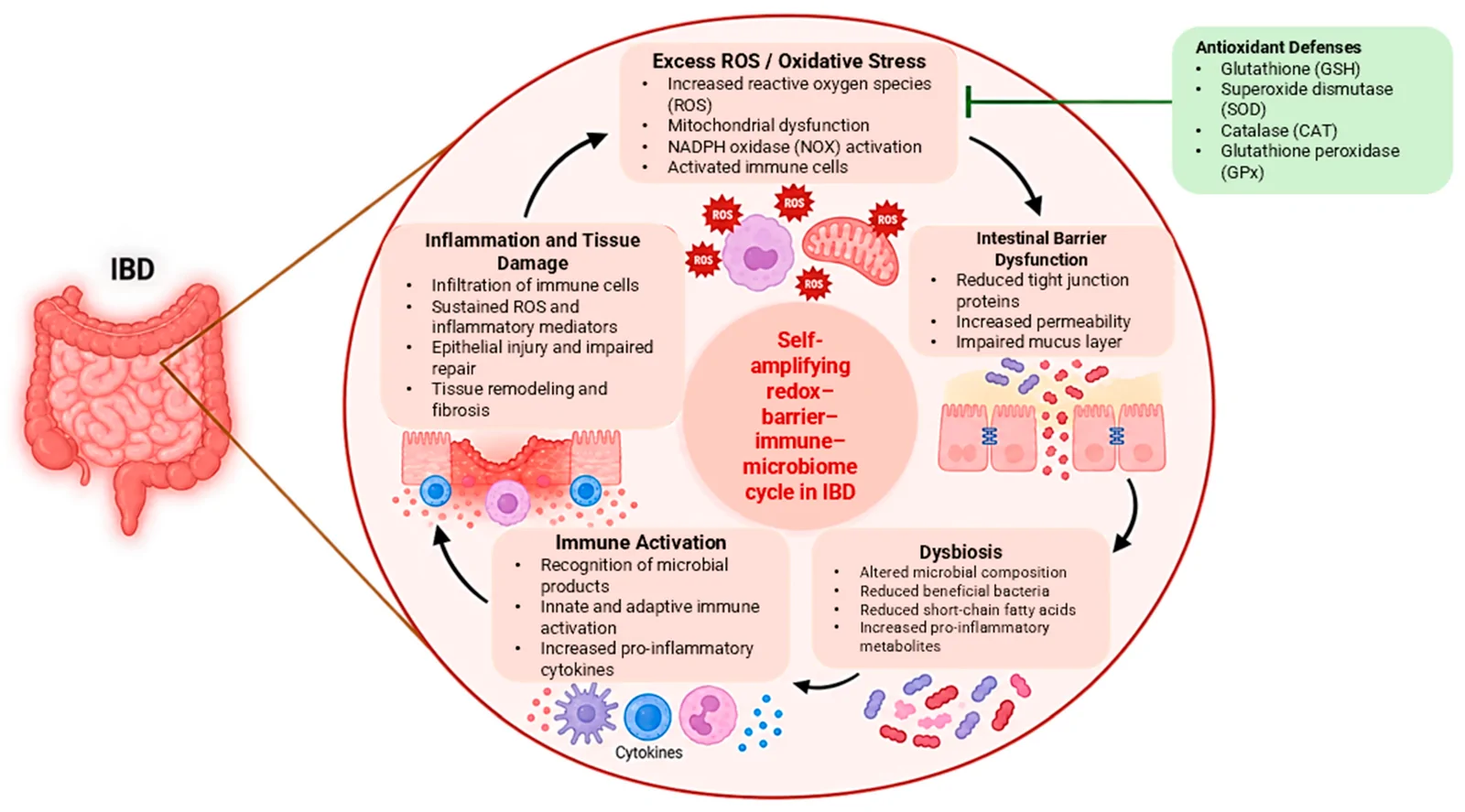
Self-amplifying redox–barrier–immune–microbiome cycle in inflammatory bowel disease (IBD). The schematic illustrates the interconnected relationships among oxidative stress, epithelial barrier dysfunction, dysbiosis, immune activation, and inflammation.

ESM studies are therefore most informative when they combine redox measurements with epithelial function, inflammatory outcomes, microbial composition, and metabolites. Changes across these domains may be coordinated without defining their temporal or causal sequence. Figure 2 summarizes the general biological relationships used to interpret these outcomes.

### 3.2. IBD as a Model of Disrupted Intestinal Homeostasis

IBD includes Crohn’s disease and ulcerative colitis, which involve interacting genetic, environmental, epithelial, immune, and microbial factors [86,87,88]. It is used here as a mechanistic model because these processes overlap with the proposed intestinal effects of ESM.

#### 3.2.1. Relevance and Limits of Colitis Models

Ulcerative colitis mainly affects the colonic mucosa, whereas Crohn’s disease can involve other gastrointestinal regions and transmural injury [86,87]. Most ESM intestinal-disease studies use mouse colitis models [11,13,22]. These models can test responses to mucosal injury or inflammation but do not represent the full range of human IBD or establish efficacy in patients. DSS-induced injury and IL-10 deficiency also represent different experimental settings: one emphasizes chemically induced epithelial injury, whereas the other involves altered immune regulation [11,13]. Agreement across models supports further investigation but should not erase these differences in disease initiation or exposure.

#### 3.2.2. Immune and Barrier Dysregulation

Microbial sensing, cytokine signaling, and epithelial defense contribute to IBD, with TNF-α, IL-1β, IL-6, and other mediators involved in mucosal injury [86,89,90,91,92]. These pathways provide relevant endpoints for ESM experiments. In ulcerative colitis, neutrophil infiltration, crypt injury, and mucin depletion connect inflammatory activity with structural barrier damage [87,91,92]. Accordingly, inflammatory-marker changes are most informative when accompanied by histological, barrier, or disease-severity outcomes.

#### 3.2.3. Oxidative Stress, Mitochondrial Dysfunction and Mucosal Injury

Oxidative damage and altered antioxidant status have been reported in IBD, with variation among markers and patients [93,94]. Mitochondrial dysfunction can link altered energy metabolism with ROS production and epithelial injury [85,95,96]. For ESM, these observations support measuring oxidative injury together with epithelial and mitochondrial function. Reported redox markers include SOD, catalase, glutathione peroxidase, free thiols, and total antioxidant capacity [93,94], which provide complementary rather than interchangeable information about cellular redox status.

#### 3.2.4. Current Therapy, Antioxidant Strategies and the Translational Niche for ESM

Established IBD treatments target inflammation and disease activity, while nutritional approaches are used in defined clinical contexts [97,98,99]. Experience with other antioxidant interventions, including variable curcumin trial results, also shows that formulation and dose can influence biological and clinical responses [100,101,102]. These observations provide a rationale for evaluating ESM-derived preparations in intestinal inflammatory settings where redox imbalance, epithelial injury, and inflammatory signaling coexist. The physiological roles of ROS further support an emphasis on restoration of redox homeostasis rather than nonspecific suppression of ROS [103].

### 3.3. Nutritional Modulation of Intestinal Redox Homeostasis

Dietary substrates influence microbial activity and host responses. Fiber deprivation can promote microbial use of mucus glycans, while responses to fiber-rich or fermented-food interventions vary among individuals [104,105,106,107,108]. For ESM, these findings emphasize the importance of considering composition, digestion, dose, background diet, and baseline microbiota when interpreting intestinal responses.

Food-derived bioactives can act through direct chemical antioxidant activity, cellular signaling, or microbial metabolism [109,110,111,112,113,114,115,116,117]. These routes are relevant to ESM because proteins and peptides that escape small-intestinal digestion may undergo microbial metabolism, producing SCFAs, branched-chain fatty acids, ammonia, and sulfur-containing metabolites. The balance of these products depends on substrate composition and microbial context, making combined measurements of ESM digestion, metabolites, and host responses particularly informative.

Individual amino acids and micronutrients can also influence epithelial, immune, and antioxidant pathways [118,119,120,121,122,123,124,125,126,127,128,129]. These broader nutritional mechanisms identify relevant endpoints, but the ESM-specific evidence considered in Section 4 is needed to determine which components and exposures are responsible for the reported intestinal effects.

## 4. Experimental Evidence for ESM in Gut Health

### 4.1. Chemical Antioxidant Assays and Cellular Redox Evidence

Chemical assays demonstrate antioxidant potential; however, cellular assays are necessary to determine if antioxidant effects translate to changes in a biological system. DPPH and ABTS/TEAC primarily assess radical-scavenging capacity, FRAP measures reducing capacity, and metal-chelation assays assess the ability to bind pro-oxidant metal ions [130,131]. A higher TEAC, FRAP, or DPPH response cannot be assumed to translate proportionally into intestinal efficacy because digestion, cellular exposure, metabolism, and host responses are not fully captured by cell-free assays [130,131].

Natural ESM and ESM hydrolysates show antioxidant activity in radical-scavenging, reducing, and metal-chelation assays, and ESM-derived peptides protect Caco-2 intestinal epithelial cells from hydrogen peroxide-induced oxidative stress [10,16,21]. However, the antioxidant activity differs among peptide fractions. For example, in the Alcalase-Protease S hydrolysates, the <5 kDa fraction showed the strongest DPPH and hydroxyl-radical scavenging activity, with IC_50_ values of 0.60 and 0.34 mg/mL, respectively, while the >10 kDa fraction showed the strongest Fe^2+^ chelating activity, with an IC_50_ of 1.80 mg/mL [16]. This suggests that molecular weight and composition influence antioxidant capacity, as peptide size can affect accessibility and interactions with reactive species, while specific amino acid residues can contribute to radical scavenging, electron donation, or metal-ion chelation [16,21].

Processing, specifically hydrolysis, influences these responses. In the 2026 Jet-O-mized ESM study, hydrolyzed fractions of ESM showed greater antioxidant capacity than the non-hydrolyzed material, indicating that, in this preparation, the measured antioxidant activity was strongly influenced by processing [18]. At 10 mg/mL, the soluble hydrolyzed fraction showed a TEAC value of approximately 667 μM Trolox equivalents, compared with approximately 43 μM for the non-hydrolyzed material [18].

ESM also affects endogenous antioxidant defenses in cellular models. Natural ESM and its hydrolysates have been associated with increased SOD-related responses and reduced H_2_O_2_-induced ROS in stressed human chondrocytes [10,21]. These findings support the cellular redox activity of ESM, although chondrocyte models provide only indirect evidence for intestinal effects.

ESM-derived peptides also affected the glutathione antioxidant system in hydrogen-peroxide-stressed Caco-2 cells. The <5 kDa peptide fraction increased intracellular glutathione and the activities of glutathione peroxidase, glutathione reductase, and glutathione S-transferase. It also increased γ-glutamylcysteine synthetase activity and expression and increased the GSH/GSSG ratio, while catalase activity was not significantly altered [12]. These changes indicate improved glutathione synthesis, recycling, and utilization, consistent with a stronger intracellular antioxidant response.

Together, these findings show that ESM-derived components can support cellular antioxidant defenses in addition to directly scavenging reactive species. Defining the responsible peptides and signaling pathways is an important next step, particularly for intestinal epithelial models.

### 4.2. Intestinal Epithelial and Immune-Cell Evidence

In a DSS-induced colitis model, dietary ESM promoted epithelial proliferation, restitution, and re-epithelialization of injured regions [11]. In the same study, ESM was associated with changes in genes related to epithelial proliferation and tissue remodeling, including increased CTGF, PDGFA, TIMP2, and TIMP3 and reduced MMP7 and MMP9 [11]. Hydrolyzed Jet-O-mized ESM also increased TEER at lower concentrations in Caco-2 monolayers, while FITC-dextran permeability remained unchanged [18]. At 1.25 mg/mL, the soluble hydrolyzed fraction increased TEER by 79.6 Ω·cm^2^ over 24 h, compared with an increase of 28 ± 10 Ω·cm^2^ in untreated Caco-2 monolayers [18]. Together, these studies provide complementary evidence for epithelial responses under direct cellular exposure and within an injured intestinal environment.

ESM has also altered inflammatory responses in intestinal epithelial cells. ESM hydrolysates reduced IL-8 secretion in stimulated Caco-2 cells, with the AL-PS hydrolysate and the <5 kDa and 5–10 kDa fractions producing significant reductions at 0.1 and 0.5 mg/mL [16]. More recent evidence showed that soluble ESM also affected inflammatory responses in LPS-stimulated Caco-2 cells. At 0.01%, soluble ESM reduced TNF-α, IL-1α, and IL-1β gene expression by 55.1%, 86.5%, and 64.3%, respectively. However, these effects were not consistent across concentrations or cytokines, as 0.1% ESM reduced IL-1α but not TNF-α or IL-1β, while IL-6 expression increased [132]. Together, these findings suggest that ESM affects inflammatory responses in intestinal epithelial cells, with different responses according to the ESM preparation, concentration, and inflammatory mediator examined.

Hydrolyzed Jet-O-mized ESM also reduced LPS-induced nitric oxide production in RAW 264.7 macrophages in a concentration-dependent manner, with up to approximately 80% inhibition depending on the formulation and concentration tested [18]. In a Caco-2/RAW 264.7 co-culture model, soluble ESM protein delivered through chitosan/fucoidan nanoparticles reduced LPS-induced NO, TNF-α, and IL-6 production and helped preserve epithelial barrier function [40]. The carrier itself also showed biological activity, emphasizing the importance of blank-carrier controls when evaluating formulated ESM.

Macrophage and epithelial–immune co-culture studies therefore extend the evidence beyond epithelial cells alone and support coordinated effects on inflammatory and barrier responses under controlled conditions.

Taken together, the cellular studies demonstrate reproducible ESM-associated effects on redox status, epithelial function, and inflammatory signaling. Whether these responses are driven by shared components or by distinct interacting pathways after oral intake remains to be resolved.

### 4.3. Animal Intestinal-Disease Models

Hydrolyzed ESM has also been tested in animal models of intestinal inflammation. In DSS-treated mice, oral ESM hydrolysates at 200 mg/kg body weight per day for 14 days reduced weight loss, clinical signs of colitis, and IL-6 levels [22]. These findings extend the cellular evidence to an integrated intestinal model and support direct comparison of hydrolyzed preparations with the native and fine ESM forms used in other studies.

Unhydrolyzed or fine ESM has shown beneficial effects in DSS and IL-10 knockout models [11,13]. In the DSS model, an 8% fine ESM diet reduced disease activity and colon shortening, while also improving epithelial repair and reducing inflammation [11]. In IL-10 knockout mice, long-term dietary ESM improved survival and was associated with lower intestinal inflammation and changes in the gut microbiota [13]. Additional work in the IL-10 knockout model also reported lower colonic inflammatory markers and changes in immune responses after ESM supplementation [78].

More recent studies have reported similar effects. In a 2025 DSS-colitis study, oral ESM pretreatment reduced disease activity, including body weight loss, diarrhea, and fecal bleeding. Histological damage to the intestinal mucosa was also reduced, although colon length was not significantly improved [133].

In an AOM/DSS model of chronic colonic inflammation, mice received an 8% ESM diet starting one month before AOM/DSS treatment and continuing throughout the study. More than 50% of control mice died by week 10, compared with 90% survival in the ESM group. ESM also reduced body weight loss, improved stool consistency, reduced rectal bleeding, and lowered disease activity scores [134]. The same study reported lower colonic IL-6 expression and lower expression of M1-like macrophage markers, including S100A8, SAA3, and NOS2. Claudin-1 expression was also higher in ESM-fed mice, while OCLN and TJP1 were not significantly changed. However, ESM did not significantly reduce colon shortening, histological inflammatory infiltration, or tumor development [134].

Together, animal studies show that ESM preparations improve selected intestinal outcomes across several inflammatory models. The exposures used, including 8% dietary ESM and 200 mg/kg/day hydrolysate, are substantially different from fixed-dose human supplementation and should not be interpreted as human-equivalent efficacy doses [11,22,134]. Section 6.3 places these exposures in the context of available human dosing data.

### 4.4. Microbiome and Metabolite Evidence

Studies have reported changes in the gut microbiota following dietary ESM supplementation. In studies using IL-10 knockout mice fed an 8% ESM diet, ESM increased microbial diversity and altered the abundance of several bacterial groups. Moreover, reported changes included increased *Ruminococcus* and Bacteroidales S24-7, reduced Enterobacteriaceae, and a shift in the Firmicutes/Bacteroidetes ratio toward that observed in control mice [13]. ESM supplementation also increased total cecal organic acids, with butyrate increasing approximately 2.3-fold [78].

Similar findings were reported in aged mice, where an 8% ESM diet for 24 weeks increased the abundance of butyrate- and valerate-producing bacteria. Cecal isobutyrate, isovalerate, and valerate increased by approximately 97%, 85%, and 98%, respectively, while total SCFA concentration did not change [79]. This provides additional evidence that ESM-related changes in microbiota occur together with measurable changes in microbial metabolites.

DSS-colitis studies have likewise reported improved microbial diversity and reduced Enterobacteriaceae and *E. coli* together with epithelial and inflammatory improvement [11]. The concurrence of these changes supports an integrated microbiome–host response and motivates experiments designed to determine their causal relationships.

Recent work has also demonstrated microbiota changes during ESM supplementation in AOM/DSS-associated colonic inflammation. ESM did not significantly change alpha diversity, but beta-diversity analysis showed that the microbial community of ESM-fed AOM/DSS mice shifted closer to that of control mice. ESM also reduced Escherichia–Shigella, increased Firmicutes and Muribaculaceae, and shifted predicted microbial metabolic pathways toward the control profile [134]. Because these functional pathways were inferred from 16S rRNA data [135], direct metabolomic or metagenomic measurements would strengthen their interpretation.

Collectively, these studies show that dietary ESM can alter microbial community structure and, in some models, microbial metabolites. Changes in butyrate and other organic acids are especially informative because they link microbial activity to epithelial and immune physiology [78,79]. Microbial diversity, taxonomic ratios, or individual taxa are less informative when considered alone.

Moreover, micronized ESM has shown lower digestibility than an ESM peptide hydrolysate under simulated gastrointestinal conditions, while also affecting microbial diversity and inflammatory outcomes in aged mice. In these mice, an 8% ESM diet increased gut microbial diversity and altered microbial composition compared with the aged control group. In vitro digestion also showed that ESM powder was less digestible than an ESM peptide hydrolysate, specifically under conditions designed to simulate gastrointestinal digestion in older adults [19].

The parallel findings on preparation-dependent digestion, microbiota, metabolites, and host responses provide a plausible basis for microbial involvement in ESM activity. Direct comparisons of gastrointestinal fate and mechanistic studies that manipulate the microbiota are now needed to determine which ESM components reach the distal intestine and whether microbial metabolism contributes to the host response.

### 4.5. Human Evidence

Human ESM studies have mainly examined musculoskeletal outcomes. Randomized trials and evidence syntheses have reported improvements in pain, stiffness, strength, or mobility with selected ESM preparations [136,137,138]. Oral doses in these studies have generally been around 300–500 mg/day [136,137,139]. These studies support the oral tolerability of ESM, but they do not provide evidence for intestinal antioxidant effects, barrier protection, microbiome-related benefits, or efficacy in IBD.

Human evidence related specifically to gut health is still limited. A 2025 study included mouse pharmacokinetic and DSS-colitis experiments as well as a double-blind randomized trial in 20 healthy adults who received ESM or a control for 8 weeks [133]. Microbiota changes were assessed using fecal samples collected before supplementation and at several time points during the intervention. Shannon–Wiener alpha diversity increased in the ESM group at weeks 1 and 8 relative to baseline, while no significant change was reported in the control group. The ESM group also showed lower relative abundance of *Bacteroides* and higher proportions of *Bifidobacterium* and Lactobacillales at weeks 4 and 8 [133].

The study had several limitations. The number of participants was small, and the modified T-RFLP method used for microbiota analysis examined a limited range of bacterial groups. Fewer participants were also included in the final microbiota analyses than were initially randomized, with 10 participants in the control group and 7–8 in the ESM group depending on the analysis [133].

These findings provide preliminary human evidence that oral ESM supplementation can alter fecal microbiota in healthy adults. The study did not assess intestinal barrier function, oxidative stress, inflammatory disease activity, or clinical outcomes in IBD. Its principal contribution is evidence of a human microbiome response rather than clinical efficacy. The experimental evidence across ESM preparations and model systems is summarized in Table 2.

### 4.6. Interpreting the Strength of ESM Evidence

The ESM literature includes chemical antioxidant assays, cell-based experiments, animal models, and limited human research. Together, these studies provide consistent evidence for antioxidant and cellular redox activity and repeated evidence for epithelial, inflammatory, and microbiome-related responses in animal models. The strength and interpretation of each finding depend on the preparation tested and the experimental system.

Hydrolyzed ESM has been studied particularly for peptide release, antioxidant activity, and direct cellular responses, whereas native or micronized ESM has been used more often in animal studies of intestinal inflammation and microbiota [11,13,16,18,19,22]. This distribution highlights distinct experimental strengths but does not establish fixed preparation-specific mechanisms. Head-to-head comparisons remain limited.

Recent studies are especially relevant to gastrointestinal translation. The 2025 human microbiota study [133] and 2026 studies of Jet-O-mized ESM processing [18], AOM/DSS-associated colonic inflammation [134], and ESM peptide nanoparticle stability [35] add information on gastrointestinal exposure, intestinal outcomes, microbiome responses, and delivery. Earlier musculoskeletal and non-intestinal studies remain useful for defining antioxidant activity, systemic responses, and oral tolerability.

Understanding how these responses relate to specific preparations requires consideration of processing, which influences material properties and shape gastrointestinal behavior. However, further research is needed to identify the active components, determine their regional intestinal exposure, establish the optimal preparation and dose, clarify causal relationships among host and microbial responses, and assess clinical relevance in humans.

## 5. Mechanistic Model of ESM Action

**Figure 3:**
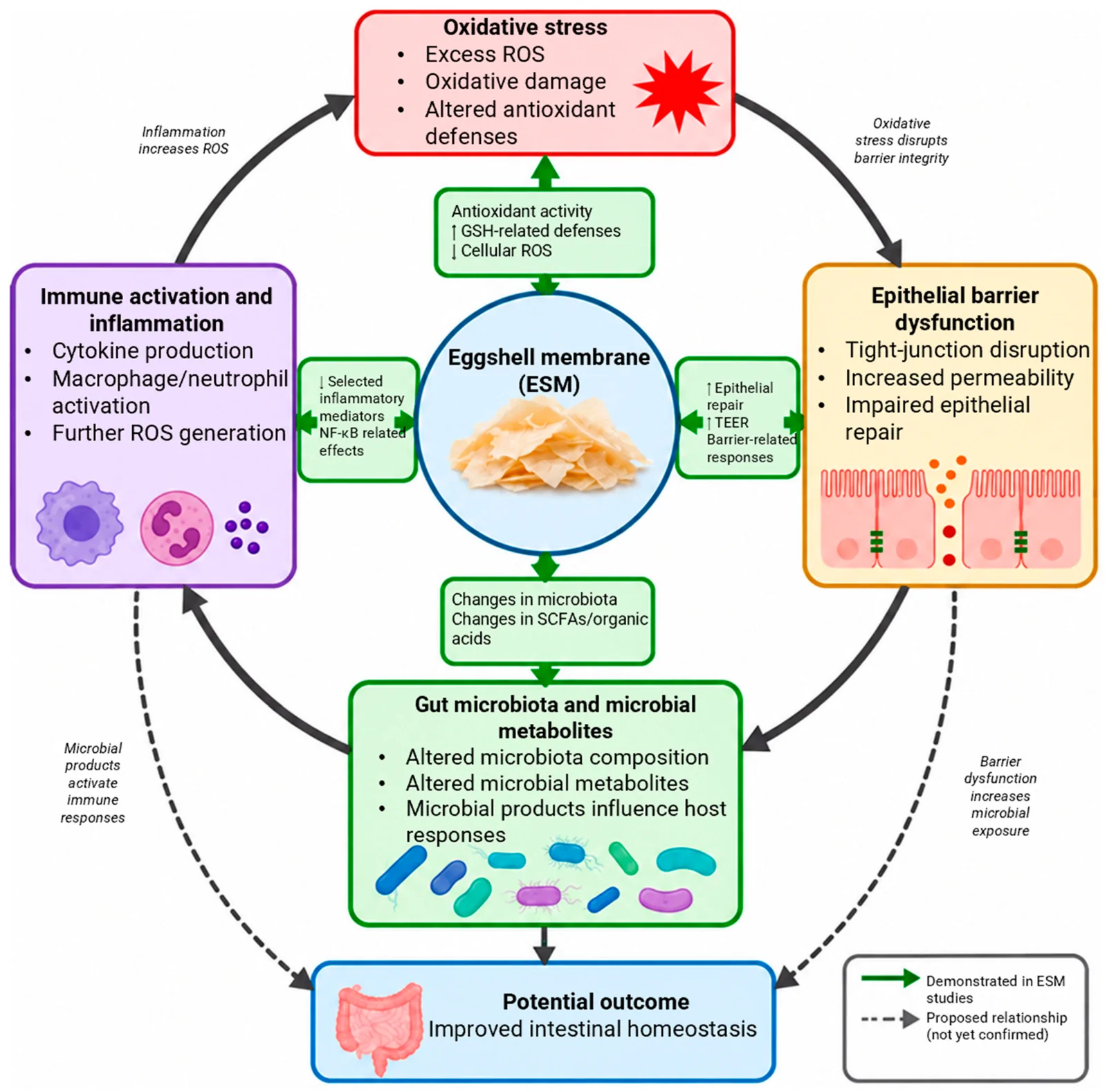
Integrated model of ESM-associated intestinal responses. ESM studies have reported effects on redox balance, epithelial barrier function, inflammatory signaling, and the microbiota across different preparations and experimental systems. Solid arrows summarize established biological relationships among these domains, whereas dashed arrows indicate proposed links in the ESM response model. Direct NF-κB effects have been demonstrated in non-intestinal cell models; their contribution to intestinal responses after oral ESM remains to be determined.

The available evidence supports an integrated model in which ESM-derived peptides and other components can influence intestinal redox balance, epithelial repair, inflammatory signaling, and microbial metabolism. Processing is likely to influence which components become available during gastrointestinal transit and therefore their relative contribution to these responses. The pathways are not mutually exclusive, and their causal relationships remain to be established. Figure 3 distinguishes experimentally reported ESM responses from the biological relationships proposed to connect them.

Hydrolysis releases smaller peptides with antioxidant activity. Lower-molecular-weight ESM peptide fractions show strong radical-scavenging activity, while metal-chelating activity varies with preparation and peptide fraction [16,21]. In Caco-2 cells, ESM-derived peptides reduce oxidative stress and increase glutathione and glutathione-related enzyme activity [12,16]. Individual antioxidant peptides, including KPLCPP and LWNPR, also affect Keap1–Nrf2 signaling in RAW 264.7 cells [33]. Together, these findings support both direct antioxidant activity and modulation of endogenous antioxidant defenses; intestinal confirmation of specific signaling pathways remains a priority.

ESM preparations also produce epithelial and inflammatory responses. Hydrolyzed Jet-O-mized ESM increases TEER in Caco-2 monolayers [18], dietary ESM promotes epithelial proliferation and repair in DSS-treated mice [11], and several preparations reduce inflammatory mediators in epithelial cells, macrophages, or epithelial–immune co-cultures [16,18,40]. These findings place barrier repair and inflammatory signaling alongside redox activity as recurring components of the ESM response profile.

Direct effects on NF-κB signaling have been reported in non-intestinal models. Processed ESM reduced overall NF-κB activity in LPS-stimulated human monocytes and altered p65/p50 signaling in macrophage-like cells, while also affecting IL-10, IL-1β, and IL-6 secretion [140]. ESM peptides also inhibited NF-κB p65 nuclear translocation and reduced inflammatory mediators in IL-1β-stimulated canine chondrocytes [141]. These studies identify NF-κB as a plausible mechanistic target for direct testing in intestinal models.

A complementary microbial route is supported by evidence that some ESM material resists gastrointestinal digestion and that dietary ESM alters microbiota composition and cecal organic acids [13,17,19,78,79,134]. Lower fecal nitrogen-based digestibility of ESM-P than that of casein and reduced small-peptide release from micronized ESM relative to an ESM hydrolysate are consistent with residual material remaining available for distal intestinal interactions [17,19]. The specific substrates reaching the colon and their microbial utilization have not yet been directly defined.

Butyrate and other microbial metabolites can influence epithelial and immune function, providing a biologically plausible connection between the microbial changes and host outcomes observed after ESM supplementation. Testing this connection will require experiments that combine preparation-specific gastrointestinal fate with metabolite measurements and direct manipulation of the microbiota.

Thus, the working model includes both direct interactions of ESM-derived components with host cells and indirect effects involving microbial metabolism. Their relative contributions are likely to depend on processing, digestion, dose, and intestinal context, providing a testable framework for future mechanistic studies.

## 6. Translational Context, Gaps and Research Roadmap

### 6.1. ESM Compared with Other Nutraceutical Approaches

Several dietary and nutraceutical approaches have been studied for intestinal health, including fermented foods, dietary fiber, polyphenols, omega-3 fatty acids, and bioactive polysaccharides and oligosaccharides [107,142,143,144,145]. Like these interventions, ESM can influence redox status, epithelial function, inflammatory responses, and the microbiota. Its distinguishing feature is the marked preparation dependence introduced by milling, hydrolysis, fractionation, and formulation. Meaningful comparison with other nutraceuticals therefore requires preparation-specific characterization and matched biological endpoints.

### 6.2. Multi-Omics and Causal Assessment of ESM Responses

Different omics approaches provide different information about how ESM affects the gut. Metagenomics identifies microbial composition and functional potential, with shotgun sequencing providing greater species- and gene-level resolution than 16S rRNA profiling [72,146]. Metatranscriptomics shows which microbial genes are actively expressed, while metabolomics measures compounds derived from the diet or produced by the host and microbiota [147,148,149,150]. Looking at these measurements together provides more information than changes in bacterial abundance alone.

Integrating multi-omics is important for ESM because several studies have reported changes in the gut microbiota, but changes in bacterial abundance do not explain what those bacteria are doing. Predictions based on 16S data suggest possible changes in microbial function, but they do not directly measure microbial genes, gene expression, or metabolites [135]. Shotgun metagenomics could identify changes in microbial pathways, while metatranscriptomics could show if those pathways are active. Metabolomics could then determine if these changes are accompanied by altered SCFAs or other microbial metabolites.

Multi-omics also allows microbial changes to be examined together with host responses. Studies could measure microbiota composition together with SCFAs, intestinal barrier markers, inflammatory mediators, and redox markers. Similar approaches have been used in IBD to examine changes across microbial, metabolic, and host responses over time [26,149,151]. For ESM, integrating these measurements would help identify which microbial changes are associated with changes in intestinal function rather than interpreting the microbiota alone.

These analyses identify associations, while causal experiments are needed to determine directionality. Studies of individual responses to dietary intake, dietary-fiber-dependent recovery of the microbiome and metabolome, and genome-scale microbial metabolic reconstruction can inform future ESM studies by highlighting interindividual variability, the influence of background diet, and microbial metabolic potential [152,153,154].

### 6.3. Translational Positioning of ESM in IBD

Dietary interventions are used in IBD, especially in Crohn’s disease, although responses vary [155,156,157]. In the absence of clinical efficacy studies in patients with IBD, ESM is best positioned as a candidate for further investigation as a supportive nutritional approach. This defines a research direction rather than a recommendation for current clinical use.

Available human musculoskeletal studies provide useful dosing examples: 300 or 500 mg/day of an ESM preparation [137], 450 mg/day of a water-soluble ESM hydrolysate [139], and 500 mg/day of NEM [136]. Selected mouse intestinal studies used 8% ESM diets or 200 mg/kg/day hydrolysate [11,22,134]. Because the preparations, species, and dosing paradigms differ, these values should not be interpreted as human-equivalent intestinal doses; the effective human intestinal dose remains unknown. Dose-ranging studies with defined preparations and intestinal endpoints are therefore needed.

Early human studies should focus on outcomes that show where ESM is having an effect. Intestinal permeability provides a measure of barrier function, which is relevant because barrier defects are common in IBD [48]. Fecal calprotectin provides a non-invasive marker of intestinal inflammation, while CRP provides information about systemic inflammation [99,158]. These markers are useful because symptoms alone do not always reflect the level of intestinal inflammation. Validated symptom scores should still be included to determine if biological changes are also associated with improvements experienced by patients [99,158].

Stool metagenomics and measurements of SCFAs or other microbial metabolites would help determine if the microbiota changes reported in ESM studies also occur in humans and if they are accompanied by changes in microbial function [26,78,79,149,151]. Safety and tolerability should also be monitored because current safety data apply to specific ESM preparations rather than all forms of ESM [41,42]. Studies in healthy adults provide information about tolerability and biological responses, but studies in patients with IBD will be needed to determine if ESM has any clinical benefit.

### 6.4. Research Roadmap

Future ESM research should begin with better characterization of the material being tested. The source of ESM, processing method, degree of hydrolysis, molecular-weight distribution, peptide and protein composition, GAG content, contaminants, and batch variability should all be reported. Such characterization is especially important because different ESM preparations behave differently during digestion and are not expected to produce identical biological effects.

Once the preparation is defined, its gastrointestinal fate should be examined more closely. Studies should determine how much of the material is digested, which peptides or other components are released, how stable they remain during gastrointestinal transit, and what reaches the distal intestine. Characterizing gastrointestinal fate would help connect the processing method with the biological effects observed later in the gut.

Mechanistic studies should then focus on the pathways and outcomes already suggested by current ESM research. These include glutathione metabolism, Nrf2, NF-κB, epithelial repair, tight-junction regulation, microbial metabolism, and SCFA profiles. Direct antioxidant activity should also be separated from changes in the cell’s own antioxidant defenses. Comparing native, micronized, and hydrolyzed ESM directly would help determine how processing changes these responses.

The role of the microbiota also needs to be tested more directly. Multi-omics identifies microbial, metabolic, and host changes that occur together, but additional models are needed to determine how they are connected. Germ-free or antibiotic-treated animals, fecal microbiota transfer, defined microbial communities, organoids, and epithelial–immune co-culture models would help differentiate microbiota-dependent effects from direct effects on host cells.

Early human studies should use well-characterized ESM preparations and dose ranges informed by preclinical exposure, preparation-specific safety data, and available human tolerability evidence. Initial trials should examine safety, tolerability, intestinal permeability, inflammatory markers, and microbiome/metabolite responses before progressing to clinical efficacy studies in gastrointestinal disease populations.

### 6.5. Personalized Response as a Later Translational Priority

Variation in the gut microbiome and other host factors could contribute to differences in individual responses to ESM. Similar differences have been observed with dietary and microbiome-based interventions [159,160]. For ESM, baseline microbiome and metabolite profiles could be examined as possible factors influencing response, but larger studies are needed before using them to identify likely responders.

### 6.6. Preliminary Intellectual-Property Considerations

Selected patent publications illustrate commercial interest in ESM processing and proposed oral or intestinal applications [161,162,163]. They are presented as examples rather than as a systematic patent landscape.

## 7. Limitations

Three limitations are particularly important when interpreting the current literature. First, ESM preparations differ in source, purification, particle size, hydrolysis method, molecular-weight distribution, peptide composition, and dose. These differences can alter solubility, digestibility, biological activity, and microbial accessibility, while direct head-to-head comparisons remain scarce.

Second, most intestinal evidence is preclinical. Cell models define direct epithelial or immune responses and animal models integrate injury, inflammation, and microbial changes, but the active ESM components, their gastrointestinal fate, regional exposure, and the causal sequence linking redox, barrier, inflammatory, and microbial responses remain incompletely defined.

Third, human intestinal evidence remains limited. Existing oral studies provide useful information on exposure and tolerability of selected preparations, and one small study reports changes in fecal microbiota in healthy adults [133,136,137,138,139]. Controlled studies with intestinal functional endpoints are needed to establish an effective dose and determine whether the preclinical response profile translates into clinically meaningful benefit.

## 8. Conclusions

Preclinical studies show that ESM preparations can enhance cellular antioxidant defenses, promote epithelial repair, modulate inflammatory responses, and alter microbial composition and metabolites. These findings support further development and evaluation of ESM as a bioactive ingredient for intestinal health. The central contribution of this review is to connect these effects with preparation-dependent material properties and gastrointestinal fate, providing an integrated redox–barrier–immune–microbiome framework for ESM research. The next priorities are direct comparison of well-characterized preparations, identification of active components and regional exposure, causal testing of host–microbiome mechanisms, and preparation-specific dose-ranging in humans. Controlled human studies will determine whether these reproducible preclinical effects translate into clinically meaningful gut-health benefits.

## Figures and Tables

**Table 1:** Processing-Dependent Properties and Gastrointestinal Relevance of ESM Preparations. Abbreviations: GI, gastrointestinal; GSH, glutathione; ROS, reactive oxygen species; MW, molecular weight. Material properties and biological responses were measured in different experimental systems, and they do not establish preferential regional intestinal exposure.

ESM Preparation	Processing	Key Material/GI Features	Reported Biological Evidence	Ref.
Native ESM	Minimal processing	Cross-linked, poorly soluble; ~87% fecal nitrogen digestibility in one rat study	Activity varies by preparation; antioxidant or intestinal responses reported in specific models	[9,10,17]
Fine/micronized ESM	Milling/particle-size reduction	Smaller physical particles; protein crosslinks remain; lower peptide generation than a hydrolysate in vitro	Epithelial repair, reduced inflammation, microbiota changes	[11,13,19]
Enzymatic hydrolysate	Alcalase, proteases or keratinase	Greater breakdown; smaller soluble peptides; composition depends on enzyme	Antioxidant activity, ↑ GSH, ↓ ROS/IL-8; improved colitis outcomes	[12,16,21,22,27]
Hydrolyzed Jet-O-mized ESM	Jet milling followed by KOH hydrolysis	Whole and soluble hydrolysates; neutralization and desalting required	Antioxidant and cellular effects in vitro; milling contribution not isolated	[18]
Peptide fractions	Hydrolysis + MW fractionation	<5, 5–10 and >10 kDa fractions show different activities	Antioxidant activity differs among peptide fractions	[12,16,33]
Commercial ESM preparations	Product-specific processing	Different solubilities; limited peptide generation in the simulated digestion tested	Solubility alone does not predict digestibility	[20]
Encapsulated ESM peptides	Carboxymethyl chitosan nanoparticles	Improved simulated GI stability; pH-responsive release	Improved antioxidant retention in vitro; in vivo advantage untested	[35]

**Table 2:** Experimental Evidence for ESM in Intestinal Health. The studies are summarized according to ESM preparation, experimental model and exposure, main findings, translational evidence stage, major limitations, and references.

ESM Preparation	Model/Exposure	Main Finding	Translational Evidence Stage	Limitations	Ref.
ESM peptide fractions/hydrolysates	H_2_O_2_- or inflammation-stimulated Caco-2 cells	↓ ROS and IL-8; ↑ GSH and glutathione-related defenses; activity differed by peptide fraction	Cellular	In vitro model; does not represent whole-intestinal physiology	[12,16]
Hydrolyzed Jet-O-mized ESM	Caco-2 cells and RAW 264.7 macrophages	↑ TEER; ↓ LPS-induced NO; effects were concentration-dependent	Cellular	Non-intestinal macrophage model; in vitro findings do not establish in vivo intestinal effects	[18]
Soluble ESM	LPS-stimulated Caco-2 cells; 0.01–0.1%	Altered inflammatory cytokine expression, with responses differing by concentration	Cellular	Responses vary by concentration and inflammatory mediator; in vitro model	[132]
ESM hydrolysate	DSS-colitis mice; 200 mg/kg/day for 14 days	↓ weight loss, clinical colitis signs and IL-6	Preclinical animal	High experimental dose; uncertain translation to human nutraceutical dosing	[22]
Fine ESM	DSS-colitis mice; 8% diet	↓ disease activity and colon shortening; ↑ epithelial repair; altered microbiota	Preclinical animal	High dietary exposure; DSS model does not reproduce all features of human IBD	[11]
Dietary ESM	IL-10 knockout mice; 8% diet	↑ survival; ↓ intestinal inflammation; altered microbiota and cecal organic acids, including ↑ butyrate	Preclinical animal	High dietary exposure; genetically driven model may limit generalizability	[13,78]
Oral ESM	DSS-colitis mice	Reduced disease activity and mucosal injury	Preclinical animal	DSS model does not reproduce all features of human intestinal disease	[133]
Oral ESM	Healthy adults; 8-week supplementation	Altered fecal microbiota	Human biological	Small sample; healthy adults; barrier function, inflammation, and IBD activity were not assessed	[133]
Dietary ESM	AOM/DSS mice; 8% diet	↑ survival; improved selected inflammatory and barrier-related outcomes; altered microbiota	Preclinical animal	High dietary exposure; not all disease-related outcomes improved	[134]
Micronized ESM	Aged mice; 8% diet for 24 weeks	↑ microbial diversity; altered microbiota and cecal organic acids	Preclinical animal	High dietary exposure; aging model rather than intestinal disease	[79]

## Data Availability

No new data were created or analyzed in this study. Data sharing is not applicable to this article.

## Abbreviations

The following abbreviations are used in this manuscript:

Abbreviation	Definition
ABTS	2,2′-Azinobis(3-ethylbenzothiazoline-6-sulfonic acid)
AOM	Azoxymethane
ATP	Adenosine triphosphate
CRP	C-reactive protein
DPPH	2,2-Diphenyl-1-picrylhydrazyl
DSS	Dextran sulfate sodium
EFSA	European Food Safety Authority
ESM	Eggshell membrane
ESM-P	Eggshell membrane protein
FITC	Fluorescein isothiocyanate
FRAP	Ferric reducing antioxidant power
GAG	Glycosaminoglycan
GI	Gastrointestinal
GIT	Gastrointestinal tract
GPx	Glutathione peroxidase
GSH	Glutathione
GSSG	Oxidized glutathione (glutathione disulfide)
HIF	Hypoxia-inducible factor
IBD	Inflammatory bowel disease
IC_50_	Half-maximal inhibitory concentration
IL	Interleukin
Keap1	Kelch-like ECH-associated protein 1
LPS	Lipopolysaccharide
MAPK	Mitogen-activated protein kinase
MW	Molecular weight
MyD88	Myeloid differentiation primary response 88
NF-κB	Nuclear factor kappa-light-chain-enhancer of activated B cells
NO	Nitric oxide
NOX1	NADPH oxidase 1
Nrf2	Nuclear factor erythroid 2-related factor 2
PPAR-γ	Peroxisome proliferator-activated receptor gamma
ROS	Reactive oxygen species
SCFA	Short-chain fatty acid(s)
SOD	Superoxide dismutase
TEAC	Trolox equivalent antioxidant capacity
TEER	Transepithelial electrical resistance
Th1	T helper 1
Th17	T helper 17
TNF-α	Tumor necrosis factor alpha
T-RFLP	Terminal restriction fragment length polymorphism
UC	Ulcerative colitis
